# Apical periodontitis associated with a calculus-like deposit: A case report of a rare fan-shaped manifestation

**DOI:** 10.1016/j.amsu.2019.03.003

**Published:** 2019-03-21

**Authors:** Kênia M. Toubes, Stéphanie Q. Tonelli, Bruno J. de Oliveira, Graziele Duarte, Eduardo Nunes, Frank F. Silveira

**Affiliations:** a Department of Dentistry, Pontifical Catholic University of Minas Gerais, Belo Horizonte, Minas Gerais, Brazil; b Dentistry Course of University of Itaúna, Itaúna, Minas Gerais, Brazil

**Keywords:** Apicoectomy, Biofilms, Cone-beam computed tomography, Dental radiography, Periapical periodontitis

## Abstract

**Introduction:**

Bacterial biofilms can be calcified. Granulomas or cystic lesions are the most commonly found entities in endodontics. Surprisingly, this case report presents a rare radiopaque image, in a fan shape, of a calculus-like deposit in the periapical region of the maxillary left central incisor.

**Case presentation:**

A 34-year-old male, with a history of trauma, presented with apical periodontitis associated with an uncommon image, similar to a calculus-like deposit adhered to the apical region of the maxillary left central incisor. Nonsurgical endodontic intervention was performed, followed by apicoectomy and histopathological analysis of the collected material. The results of the biopsy were not compatible with a cyst or granuloma but showed fibrous connective tissue with calcified areas.

**Discussion:**

Correct diagnosis in endodontics is possible with a well-conducted anamnesis, complementary imaging exams and, in some cases, histopathological analysis. The periapical calculus-like deposit, associated with a periapical radiolucent lesion, was a result of the body's fight for healing, producing unusual radiopacity.

**Conclusion:**

The presence of the calculus-like deposit in a fan shape at the root surface represented dystrophic calcification as a manifestation of the attempt to heal. In the present case, apicoectomy and tissue biopsy for histological evaluation were fundamental for the correct diagnosis.

## Highlights

• Root canal is critical area for biofilm development.
• Extraradicular biofilm on the surface of the root may cause persistent periapical lesions.
• Uncommon radiopaque image association with endodontic periapical lesions was related.
• Persistent periapical lesions associate with extraradicular biofilm represents a challenge for Endodontic treatment.
• Only surgical resection was capable to repair the injury.

## 1 Introduction

Periapical lesions are the outcome of an inflammatory reaction to an infection or traumatic injuries inside the root canal system, leading to a reduced mineral density of the affected periapical bone [[1], [2], [3], [4]]. These resorptions are usually identified as radiolucent areas on radiographic images [2]. However, the immune system has several ways to attempt to repair these lesions, varying according to the degree and duration of the injury and the quality of the immune system [[1], [2], [3], [4]].

Several studies have demonstrated the formation of a biofilm from bacterial invasion in the extraradicular area, adherent to the cementum around the root apex [3,[6], [7], [8], [9]]. Bacterial biofilms can be calcified, as the calculus name suggests [6,9]. The bacterial ability to form biofilm communities is relevant to therapeutic endodontics [6] and have been associated with apical periodontitis [2,[4], [5], [6]].

Historically, clinical examination data associated with the radiographic presentation were central to the diagnosis of apical periodontitis [[10], [11], [12], [13], [14]]. Nevertheless, the final diagnosis of these alterations can only be confirmed through histopathological analysis. In this context, a tissue collection is exclusively obtained through surgical intervention [[13], [14], [15], [16], [17]], considered a viable therapeutic alternative in endodontics.

The aim of the present study was to report a case of apical periodontitis associated with an unusual, fan-shaped, radiopaque image of the periapical region of the maxillary left central incisor in a 34-year-old male. This curious endodontic calculus-like deposit resulted from an intense inflammatory response, leading to the formation of granulation tissue with calcified areas, which demonstrates the attempt of the organism to repair itself [6,9].

## 2 Case presentation

In accordance with the principles laid down in the Declaration of Helsinki, this case report integrates a project research previously approved by Ethics Committee (CAAE: 01958818.4.0000.5137). This work is reported in line with the SCARE criteria [11].

A Brazilian patient (WAM, 34 years old, male, pheoderm) reported to the endodontic clinic of our institute, complaining about the presence of a swelling in the region of the maxillary anterior teeth. During the interview, he reported a car accident 13 years ago. The patient did not remember the dental procedures that had been performed. The patient was a strong and healthy young man, with no history of alcoholism and smoking. No signs and symptoms of chronic diseases. Your parents are alive and healthy.

**Fig. 1:**
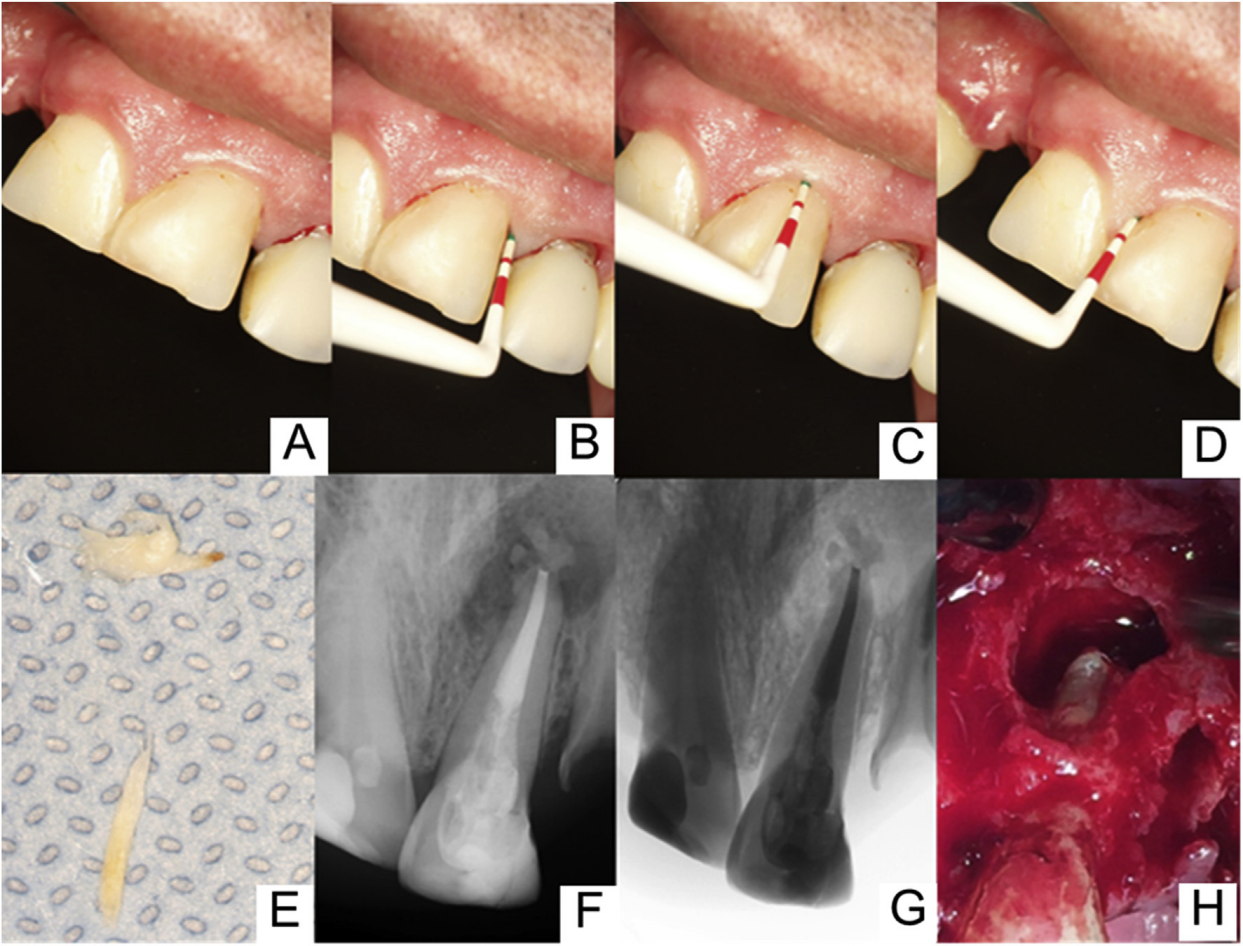
(A–D) Maxillary left central incisor showed no significant periodontal probing depth; (E) Fragments of cotton ball and cone of absorbent paper removed from canal; (F–G) Radiographic exam of final canal filled; (H) Surgical access.

On clinical examination, the presence of a labial swelling was observed in the apical region of the maxillary left central incisor. The referred element presented no significant periodontal probing depth, and endodontic access was sealed with a resin composite (Fig. 1A–D). In addition, advanced periodontal involvement was observed in the maxillary left lateral incisor and canine, whose periodontal pocket depth reached 12.0 mm at some sites. The lateral incisor crown showed considerable mobility.

**Fig. 2:**
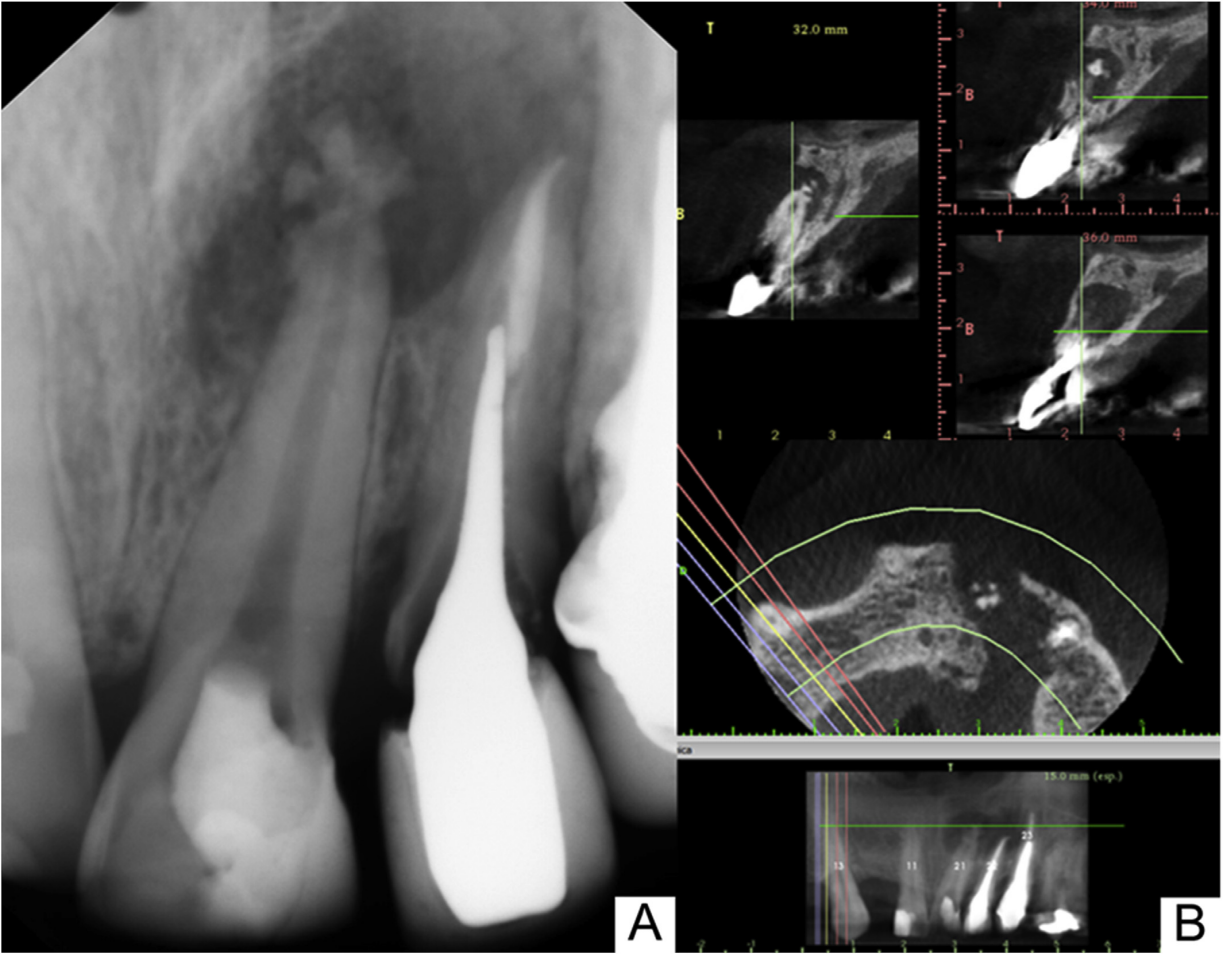
(A) Preoperative radiograph image showing fan-shaped radiopaque image associate with radiolucent area on the apex of the maxillary left central incisor extending apically to the lateral incisor, associated with radiopaque areas. (B) CBCT image showing hypodense, heterogeneous, irregular edges.

Periapical radiographic examination showed an extensive radiolucent area associated with the apex of the maxillary left central incisor, extending to the lateral incisor (Fig. 2A). The presence of a fan-shaped radiopaque image of unknown nature was noted at the apex of the central incisor. In the lateral incisor and canine, the presence of an intracanal post was observed (Fig. 2A). To better manage the case, cone-beam computed tomography (CBCT) was requested to examine the relationship of the involved teeth with the adjacent structures and the extent of the bone loss.

The CBCT images (Fig. 2B) showed a partially edentulous maxilla with the absence of tooth #12. Teeth #22 and #23 were endodontically treated and diagnosed with a vertical root fracture, and a hypodense, heterogeneous image, with irregular borders, extended from the midline to tooth #23 in the mesiodistal direction. In the apical-occlusal direction, the hypodense image extended from the apexes of teeth #21 and #22 to the middle third of the bone border. Destruction of the buccal and palatine cortical bone was also observed in the region of teeth #21 and #22. Moreover, a hyperdense mass was present inside the hypodense image, adjacent to the apex of tooth #21. The tomography report suggested a heterogeneous osteolytic lesion, with destruction of the buccal and palatine cortical bone. A final impression was compatible with several diagnostic hypotheses: (1) a calcified odontogenic cyst/calcified epithelial odontogenic tumor; (2) an odontogenic adenomatoid tumor (extrafollicular); (3) an ameloblastic fibro-odontoma; and (4) chronic osteomyelitis. Clinical, radiographic and tomographic findings were previously explained to the patient, as well treatment plan. Before the procedures, all possible discomforts and risks had been full explained the patient was confident about performance and results of the proposed treatment.

Endodontic retreatment of tooth #21 was performed in multiple sessions. Initially, the patient was administered anesthesia by an infiltration technique with 2% lidocaine with epinephrine, 1:200,000 (Alphacaine; DFL, Rio de Janeiro, RJ, Brazil), followed by isolation with a rubber dam and access to the root canal system of the central left maxillary incisor. A cotton ball and two cone fragments of absorbent paper for intracanal medication were removed from the interior canal (Fig. 1E). The pulp chamber was copiously irrigated with 5% sodium hypochlorite (Lenza Farma, Belo Horizonte, Brazil), and the canal was instrumented with a ProTaper NEXT automated system (Dentsply, Maillefer, Ballaigues, Switzerland), alternating with hand K-files second series (Dentsply, Maillefer). Subsequently, the canal was properly dried with absorbent paper cones, filled for healing with a calcium hydroxide base paste (Calen, SS White, São Paulo, Brazil), and temporarily sealed (Coltosol, Rio de Janeiro, Brazil). After 14 days, the dressing was removed, and filling with gutta-percha points (Ultimate Dental, TN, USA) and the Pulp Canal Sealer™ EWT cement (a pulp canal sealer with an extended working time; Kerr/Sybron Dental Specialties, CA, USA) was carried out (Fig. 1F and G).

**Fig. 3:**
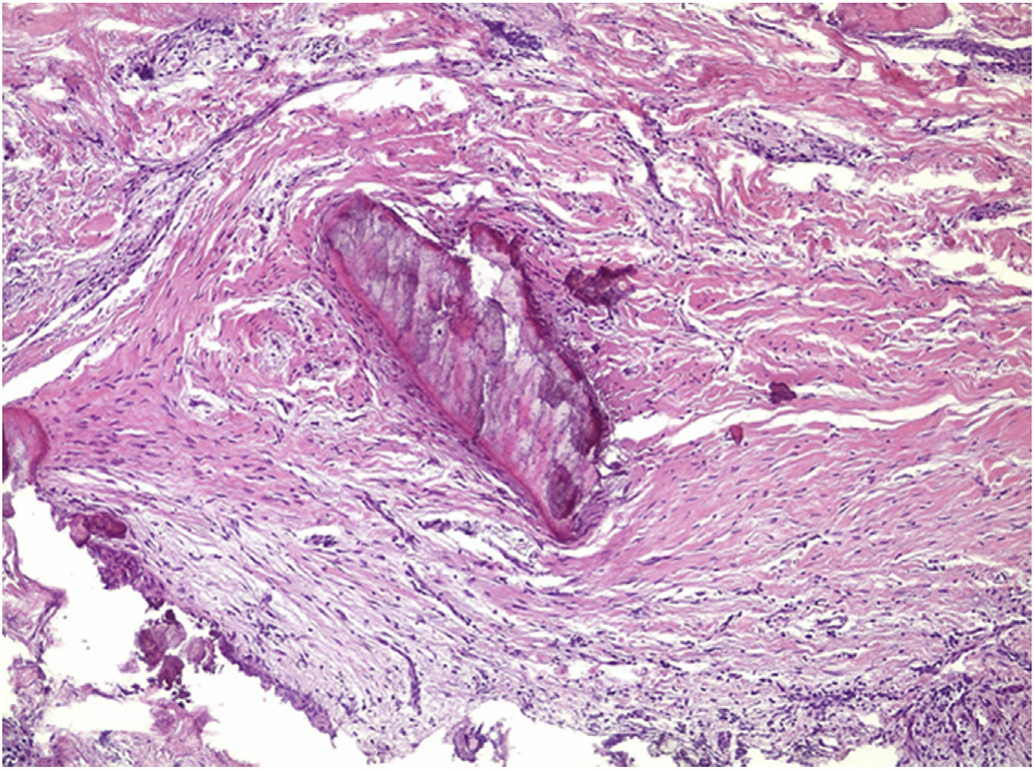
Histopathological analysis image which can be observed fibrous connective tissue presenting calcified areas.

After 7 days of nonsurgical endodontic treatment, the patient was referred to a maxillofacial surgeon with extensive experience for surgical intervention because of the extent of the bone loss and its proximity to noble areas. After surgical access, we carried out enucleation of the lesion, which was still attached to the end of the resected root (Fig. 1H). All surgical procedures were performed under a microscope for magnification. After surgery, patient was given all the necessary support post-surgery, with stitch removal and clinical control. The collected tissue was immersed in 10% formalin and sent for histopathological analysis. The results of the biopsy showed repair tissue composed of fibrous connective tissue with calcified areas (Fig. 3), suggestive of healing tissue with dystrophic calcification.

## 3 Discussion

Granulomas or cystic lesions are the most commonly found entities in endodontics [19,20]. The diagnosis of periapical lesions of inflammatory origin is still a challenge in endodontics [1,2]. Traditionally, the diagnosis of apical periodontitis is based on empirical methods, including clinical and radiographic presentations of these injuries [13,14]. Unlike common radiographic findings [[1], [2], [3], [4]], this case report presents a radiolucent area associated with the apex of the left central incisor, which also had a radiopaque image in the form of a fan of unknown nature.

CBCT, already well established in the literature, is a fundamental tool for the diagnosis and treatment planning [[1], [2], [3],[15], [16], [17], [18], [19]]. In the present study, three-dimensional evaluation was essential for determining the complexity of the treatment, based on the location and extent of the lesion.

A similar study in the literature describes another case of unusual radiopacity associated with a periapical radiolucent lesion on the root surface of the maxillary left central incisor, previously treated endodontically [9]. Pathological examination revealed calcified particles such as calculus deposits, incorporated in granulomatous tissue, which was similar to the findings in our case, except its radiographic presentation that brings an exclusive image in the form of a fan to the literature.

Surprisingly, this case presents an unusual connective tissue with dystrophic calcified areas, as an intense response of the body in an attempt to heal. Another study of a series of cases has emphasized that periapical surgery should be considered the last resort in endodontics to effectively deal with periapical lesions that result from pulp necrosis. The study showed that different extraradicular infections were associated with persistent exuding symptoms, leading to endodontic failure, and were successfully treated only by periradicular surgery [3,18]. Unfortunately, in our study, bacterial culture was not possible. The presence of highly resistant and pathogenic microorganisms might explain the degree of injury, due to an intense tissue response provoked at the apical level.

Therefore, confirmation of the final diagnosis was provided by histopathological examination of tissues, impractical in the case of nonsurgical endodontic treatment [[18], [19], [20]]. This case presents an unusual connective tissue with dystrophic calcified areas, as a result of an intense response of the body in an attempt to cicatrize. In another study, anatomopathological examination revealed calcified particles, such as calculus deposits, incorporated in granulomatous tissue, findings similar to those in the present case, except that our radiographic presentation brings an exclusive fan-shaped image to the literature.

## 4 Conclusion

This study demonstrates a case of apical periodontitis associated with a deposit similar to periapical calculus that represents an unusual response of the organism in repairing the damages caused by the presence of extraradicular biofilm and, consequently, the intense inflammatory response. The surgical approach was essential for the diagnosis and appropriate treatment of this occurrence.

## Consent

Written informed consent was obtained for publication of this case report.

## Provenance and peer review

Not commissioned externally peer reviewed.

## Ethical approval

In accordance with the principles laid down in the Declaration of Helsinki, this case report integrates a project research previously approved Ethics Committee (CAAE: 01958818.4.0000.5137).

## Sources of funding

The author affirm that did not receive any sources of funding for your research.

## Author contribution

All authors contributed significantly and in agreement with the content of the article. Tonelli, Toubes and Silveira collected all datas and photographs to draft the manuscript. Toubes realized the endodontic treatment of the canal. Oliveira realized the surgery. Tonelli, Nunes, Silveira, and Duarte wrote the manuscript for submission. All authors presented substantial contributions to the article and participated of correction and final approval of the version to be submitted.

## Conflicts of interest

The author declare none conflict of interest statement.

## Research registration number

The authors declare that studies like this have already been performed/reported in the literature.

## Guarantor

Dr. Frank Ferreira Silveira.
